# In horses with secondary sinusitis caused by dental disease, is computed tomography more accurate than radiography for the identification of apical dental pathology?

**DOI:** 10.18849/ve.v7i2.491

**Published:** 2022-04-07

**Authors:** Rebecca Gill-Parsons+, Emma Shipman+, Kirstie Pickles+

**Keywords:** APICAL DENTAL PATHOLOGY, COMPUTED TOPOGRAPHY, DENTAL DISEASE, EQUINE, HORSE, RADIOGRAPHY

## Abstract

PICO question

In adult horses with secondary sinusitis caused by dental disease, is computed tomography more accurate than radiography for the identification of apical dental pathology?

Clinical bottom line

Category of research question

Diagnosis

The number and type of study designs reviewed

The literature search identified four papers that were critically reviewed. The publications consist of two retrospective case controlled studies, one clinical study and one descriptive study

Strength of evidence

Weak

Outcomes reported

Four studies reported the sensitivity of computed tomography (CT) for the diagnosis of apical dental pathology in horses presented for evaluation of clinical signs of sinus disease with histopathological evidence of apical dental pathology. All studies reported the radiographic changes present in these horses or used absence of definitive radiographic changes consistent with apical dental disease as a reason to undertake further CT evaluation. All four papers found that CT identified teeth with apical pathology that radiography had not

Conclusion

CT is more accurate than radiography for the diagnosis of equine maxillary apical dental pathology; however, clear guidelines on the CT changes associated with apical dental pathology are required. Loss of the lamina dura, infundibular changes or pulpal gas as singular findings on CT imaging can be seen in teeth with no underlying histopathological evidence of apical disease, and in maxillary teeth imaged in horses without clinical signs of maxillary cheek tooth pathology

How to apply this evidence in practice

The application of evidence into practice should take into account multiple factors, not limited to: individual clinical expertise, patient’s circumstances and owners’ values, country, location or clinic where you work, the individual case in front of you, the availability of therapies and resources.

Knowledge Summaries are a resource to help reinforce or inform decision making. They do not override the responsibility or judgement of the practitioner to do what is best for the animal in their care.

## The evidence

The literature search identified four studies where computed tomography (CT) and radiographic findings of maxillary apical dental pathology were compared.

Two observational studies reported the accuracy of radiography and CT for diagnosis of maxillary cheek tooth apical infection confirmed by gross and histopathological findings (Liuti et al., 2018a; and 2018b).

Two retrospective case controlled studies investigated radiographic and computed tomographic changes present in maxillary cheek teeth from horses with clinical signs consistent with sinusitis (Bühler et al. 2014; and Henninger et al. 2003).

Bühler et al. (2014) reported malodourous nasal discharge, facial swelling or sinus tract formation as clinical evidence of sinusitis in all studies where clinical cases were recruited. Henninger et al. (2003) reported clinical signs of 2 weeks to 7 years duration. Bühler et al. (2014) and Liuti et al. (2018a) did not record the duration of clinical signs. The number of adult horses within these studies ranged from 18–49.

Liuti et al. (2018a) evaluated radiographic and CT findings of 29 horses with clinical signs of sinusitis. CT findings consistent with apical dental pathology included heterogeneity of the pulp, changes to pulpar volume, gas within or widening of the periapical periodontium, root clubbing and fragmentation and periapical alveolar bone lysis. Radiographic changes consistent with apical dental pathology included periapical sclerosis and apical clubbing. Histopathological evaluation was performed on the 32 teeth removed from these horses based on the CT findings. 32/32 (100%) of teeth with CT findings consistent with apical dental pathology were found to have histopathological evidence of apical dental pathology and one histologically healthy tooth having abnormal CT findings. In contrast, radiographic abnormalities were detected in only 17/32 (53%) teeth.

When a similar study design was used to evaluate cheek teeth from a cadaver population with unknown clinical histories, Liuti et al. (2018b) found 27/28 teeth (96%) with histologically identified apical pathology had CT changes consistent with apical disease, whereas radiography identified abnormalities in 14/28 teeth (50%). Henninger et al. (2003) reported 16/18 (89%) horses with clinical signs consistent with maxillary sinusitis to have CT changes consistent with apical dental pathology and only 5/18 (28%) having radiographic evidence of apical infection.

A retrospective case controlled study (Bühler et al., 2014) investigating the prevalence of apical dental pathology in horses with clinical evidence of sinusitis found 27/28 (96%) horses with inconclusive radiographic evaluation had CT changes of apical dental pathology. Three or more of the following CT changes were considered indicative of apical dental pathology; clubbing of the tooth root, widening of the periodontal space, nondetectable lamina dura, periapical sclerosis, and changes within the pulp cavity (increased pulp horn volume, irregular margination and heterogenous density) and infundibular changes (hypoattenuating occlusal surfaces, linear hypoattenuation along the infundibular length or linear attenuation with a bulbous shape at its apical extent).

An older study (Henninger et al., 2003) found CT changes consistent with apical dental pathology in 16/29 (55%) horses presented for evaluation of sinusitis, with only 5/29 (17%) horses showing radiographic evidence of apical infection. The CT findings consistent with apical dental disease included infundibular hypoattenuation, apical bulging of the tooth socket, root fragmentation and hypoattenuating gas within the tooth socket.

## Summary of the evidence

**Liuti et al. (2018a):** 

Population:	Horses (age range 3–15 years) from one hospital presented with chronic unilateral nasal discharge or maxillary swelling.
Sample size:	32 maxillary cheek teeth from 29 horses and four control cheek teeth from a cadaver.
Intervention details:	To compare the radiographic and CT findings with the gross and histological findings to assess the accuracy of each imaging technique. Initial identification of apically infected teeth from clinical examination and oral endoscopy, followed by radiographic and CT imaging. Teeth were then extracted based on the outcome of these findings. Extracted teeth underwent further CT, gross pathological and histological examinations by two independent examiners. Histological slides were anonymised and relabelled before examination and standard radiographic projections used.
Study design:	Observational clinical study.
Outcome studied:	The accuracy of radiography and CT in the detection of early apical infection.
Main Findings (relevant to PICO question)	Radiographic apical pathology was present in 17/32 (53%) of teeth. 32/32 (100%) teeth had CT evidence of apical disease with the presence of pulpar changes in all teeth. Gross pulpar abnormalities were present in the pulpar of 29/32 (91%) teeth and apical changes in 31/32 (97%) and histological pulpar changes were present in 31/32 (97%) teeth. One tooth had no detectable pulpar abnormalities, including assessment of five additional histological sections.
Limitations:	Gold standard histopathological evidence of apical dental pathology was, by study design, available from clinical cases where tooth removal was predetermined due to abnormal CT finding. Histopathology results were only available from four control maxillary teeth. The clinical history from the cadaver specimens used as controls was unknown. Specific radiographic features indicative of apical infections were decided upon consensus of highly indicative features rather than independent characteristics and same CT findings were agreed upon consensus by both examiners. Criteria of radiographic features consistent with apical dental pathology were not specified beforehand. The conclusion that later radiographic examination would likely have shown a higher proportion with definitive radiographic changes was not evidenced in this paper as the duration of clinical signs was not analysed for association with radiographic findings. CT evidence was the sole reason for the extraction of an apparently healthy tooth (as determined by gross and histological examinations) indicating a false positive – discrepancies between CT and pathology were unclear. No control sinusitis population. Poor evidence of duration of infection or clinical signs. Limited to a small number of cases and predominately descriptive in nature.

**Liuti et al. (2018b):** 

Population:	54 cadaver horse heads.
Sample size:	30 abnormal cheek teeth (26 maxillary and four mandibular) from 26 heads with gross and imaging pathological abnormalities; remaining 28 heads had no abnormalities on gross pathology and imaging and will not be commented on further in this Knowledge Summary.
Intervention details:	To compare the CT and radiographic findings with the histopathological findings. Images were evaluated by two observers with consensus obtained. Radiographic changes included root clubbing and alveolar bone sclerosis. CT changes included gas within pulps, irregular pulp horn margins, increased pulpar volume, widened periodontal space, gas within periapical tissues, root clubbing, root fragmentation, periapical halo, periapical bone changes including peripheral sclerosis, alveolar bone thickening, resorption / lysis more axially, and infundibular changes.
Study design:	Observational descriptive study.
Outcome studied:	The accuracy of CT and radiography in detecting apical infection of the cheek teeth by comparing these imaging modalities and histopathological findings.
Main Findings (relevant to PICO question)	Gross pulpar abnormalities present in 23/28 (82%) teeth and gross periodontal thickening or swelling of periapical region present in 25/28 (89%) extracted teeth. Of 28 teeth that were confirmed by histology to have apical infection, 23 had gross pulpar abnormalities, 26 had apical calcified tissue changes, and 26 had periapical periodontal changes. In all 16 teeth with severe histological alveolar bone changes, perialveolar bone changes were visible on CT, but only four of these 16 cases had identifiable periapical alveolar changes radiographically. CT imaging showed convincing evidence of apical infection on CT imaging in 27/28 teeth (96%). Radiographic abnormalities were found in 14/28 (50%) apically infected teeth.
Limitations:	One tooth with pulpar discolouration and histological evidence of apical infection did not have any CT imaging evidence of apical disease (false negative). No previous history of the horses. Images were evaluated by two observers but this was not undertaken independently. Teeth were initially screened as apically infected by imaging and / or clinical examination before being removed for histopathological evaluation.

**Bühler et al. (2014):** 

Population:	Horses (age range 4–20 years) scanned with CT at one clinic between February 2008 and June 2010.
Sample size:	1,764 roots and 1,176 infundibula of 588 upper cheek teeth from 49 horses.
Intervention details:	The 49 cases were split into two groups: Group 1 (28 horses): Horses with clinical signs suggesting primary apically infected maxillary cheek teeth (malodourous, purulent, or sanguineous nasal discharge, facial swelling, and sinus tract formation) but without obvious findings on oral examination and where radiographs were inconclusive. Group 2 (21): Horses without clinical evidence of apically infected maxillary cheek teeth (examined for unrelated reasons). CT images were observed by a board-certified radiologist and the first author. Data sheets evaluating the presence or absence of CT findings were compiled for each cheek tooth, infundibulum, and root in all cases.
Study design:	Retrospective case control study.
Outcome studied:	The prevalence and relationship of CT findings in maxillary cheek teeth in horses with and without clinical evidence of apical infection. Apical infection was diagnosed if three or more of the CT findings from: changes of the pulp cavity, clubbing of the root, widened periodontal space, nondetectable lamina dura, periapical sclerosis – these features were noted and then subjectively scored as mild, moderate or severe.
Main Findings (relevant to PICO question)	Overall, all horses had CT changes in at least one cheek tooth root – 1,499/1,764 (85%) of all investigated roots had various degrees of CT abnormalities. Nondetectable lamina dura was present as the most common single CT abnormality in 1,338 of 1,764 roots imaged. 783/1,338 (59%) roots with no detectable lamina dura were from horses with clinical evidence of apical infection and 555/1,338 (42%) roots from horses with no evidence of apical infection. CT changes more prevalent in horses in group 1: 14 roots had pulpitis in group 1 whereas only one in group 2, clubbing of the root was only present in group 1 (in 29 roots), periapical sclerosis was present in 114 roots in group 1 and six in group 2, widened periodontal space was present in 42 roots in group 1 and one in group 2, and infundibular changes found in 303 infundibula, 182 from group 1. In group 1, mild, moderate, and severe apical infection was diagnosed in 35, 19 and 14 roots and in 15, eight and 10 maxillary cheek teeth, respectively. 27/28 horses with clinical signs of maxillary tooth root pathology were confirmed by CT to have apical infection. One horse from group 2 had CT changes consistent with apical tooth root disease infection but no associated clinical signs. Radiographic changes consistent with apical dental pathology were not discussed.
Limitations:	The radiographic features that were examined initially were not specified as to what placed the horses in group 1. CT was only undertaken in horses with inconclusive changes of apical dental pathology on radiographic evaluation.

**Henninger et al. (2003):** 

Population:	Horses (ranging from 1.8–18.1 years) referred between 1998 and 2000.
Sample size:	18 horses.
Intervention details:	Referred for advanced diagnostic imaging and presenting with any of the following clinical signs: Unilateral or bilateral nasal discharge, purulent conjunctivitis, facial swelling and / or epiphora. Radiography of the head was performed before CT. Abnormal CT findings: Cavity abnormal findings of hypoattenuation. Grade I: Loss of enamel/dentine stripes, gas within the infundibulum. Grade II: Gas within the dentine / enamel border or within the pulp. Grade III: Fractured tooth. Root infection – Borderline: Apical bulging of the socket more than 10 mm off the enamel walls or irregularity. Mild: Total hypoattenuating spot (gas) within the socket or apical fragmented root. Moderate: Gas and fragmented root at the same time, increased bulging of 1–3 cm. Severe: Apical soft tissue changes >3 cm as before and secondary sinusitis, occluded nasomaxillary aperture, hyperattenuation representing a mass.
Study design:	Retrospective observational case control study.
Outcome studied:	The usefulness of CT to evaluate dental lesions occurring with nasal and / or paranasal disease. CT changes divided into cavity and root for abnormal findings which were divided into mild, moderate, and severe.
Main Findings (relevant to PICO question)	54 maxillary cheek teeth were recognised as diseased between the 18 horses, the most common tooth being 109. The entire rostral maxillary sinus was filled with material of soft tissue opacity in 17/18 horses and CT changes of the nasal cavity were identified in 16/18 horses with nasal discharge. Apical infection was identified as the underlying cause of sinusitis in 15 horses. 12/18 horses had changes in the cavity and 16/18 horses had root changes on CT evaluation but only five horses were identified as having apical pathology on radiographic imaging.
Limitations:	Older study with less clearly defined CT criteria for the presence of apical dental pathology. All horses were radiographed first, and no comparison is made between radiography and CT yet the study concludes that CT allows a more confident diagnosis over radiography. Criteria for radiographic apical changes were not defined nor was it specified if the same teeth were affected radiographically and with CT. Only 18 horses. No discussion of whether images were reviewed by one or multiple persons.

## Appraisal, application and reflection

No randomised control studies exist within the literature directly comparing CT to radiography in the diagnosis of apical dental pathology in adult horses. Three of the four papers in which radiography and CT were undertaken presented data from clinical cases presenting to veterinary hospitals and it is therefore understandable that CT was undertaken in some cases, where radiography was inconclusive and histopathological evaluation of the tooth roots could only be undertaken on teeth deemed to have apical pathology by radiographic or CT evaluation necessitating removal on patient welfare grounds.

All four papers comparing CT and radiographic evaluation of apical dental pathology either in horses with clinical signs consistent with sinusitis or in cadaver specimens found CT to identify lesions in horses without radiographic changes of apical dental disease. In all studies, the sensitivity of CT to detect apical dental pathology was greater than the reported sensitivities for radiography alone.

There is a wide range of sensitivities reported for radiographic evaluation of apical dental pathology which was likely attributed to differences in radiographic technique and variability in the criteria on which a diagnosis of apical disease was made, as discussed within the literature. Radiographic changes consistent with apical dental pathology were listed as apical blunting, periapical halo and crown fracture (Liuti et al., 2018a) root blunting and alveolar bone sclerosis (Liuti et al., 2018b). Other studies that do not directly compare CT to radiography have further stated radiographic findings that indicate criteria for dental pathology as sclerosis, cementosis, clubbing of the tooth root, interruption of the lamina dura, loss of dental density, lucency surrounding the apex of the tooth and fracture lines (Weller et al., 2001); and periapical halo formation, periapical sclerosis, clubbing of the root, loss of lamina dura and widening of the periodontal ligament (Townsend et al., 2011). The criteria used to make a radiographic diagnosis of apical dental pathology was made was not recorded in two papers (Bühler et al., 2014 and Henninger et al., 2003).

Some variations existed in reported CT changes associated with apical dental pathology. Changes were generally considered infundibular, pulpal or associated with the alveolar bone. Specific CT changes consistent with apical dental pathology were listed as increased pulpal volume, irregular pulp horn margins, periapical gas, widening of the periodontal space, root clubbing, root fragmentation, periapical halo, periapical alveolar bone lysis, dental fracture, and infundibular changes and with the addition of alveolar bone thickening and pulpal gas (Liuti 2018a; and 2018b). Infundibular gas, pulpal gas, tooth fracture, gas within the socket, apical bulging of the socket were reported (Henninger et al., 2003) and increased pulpar volume, irregular pulp horn margins and heterogeneous density, root clubbing, widening of the periodontal space >1 mm, nondetectable lamina dura, periapical sclerosis and infundibular changes (hypoattenuating occlusal surface, linear hypoattenuation along the infundibular length or linear hypoattenuation with a bulbous shape at its apical extent, and tooth fractures (Bühler et al,. 2014).

Bühler et al. (2014) found individual CT abnormalities other than clubbing of the root in dental apices of horses without clinical signs of sinusitis and loss of lamina dura in 1,338 of 1,764 (76%) of tooth roots examined with 555 roots being from the 21 horses without clinical signs of dental disease. Likewise, infundibular changes as a solitary CT feature were not significantly associated with other CT signs of apical infection. Liuti et al. (2018a) also found one tooth containing gas pockets in the apical aspect of one pulp and adjacent periodontal space where no pathological changes were found following its extraction. Single CT changes may therefore be evident in teeth from horses without clinical evidence of dental pathology and in teeth with no histopathological abnormalities and therefore loss of or nondetectable lamina and infundibular changes may be a poor indicator of apical infection as a single pathological change and may need the presence of other changes to be indicative of pathology.

The literature supports the use of CT imaging in the diagnosis of apical dental pathology in horses with signs of sinusitis as being more sensitive than radiography. In all four papers comparing CT and radiographic evaluation of apical dental pathology, CT identified lesions in horses without conclusive radiographic evidence of pathological changes consistent with apical dental disease. Consequently, all four papers reported a higher sensitivity of CT to detect pathology compared with radiography. However, care should be taken in interpreting individual CT changes, particularly loss of lamina dura, infundibular changes, and gas pockets within the pulp, as these individual changes have been reported in teeth from horses without signs of dental disease and without histopathological evidence of apical pathology. Therefore it is recommended that additional pathological changes should be identified to meet the criteria of dental apical pathology.

## Methodology Section

**Search Strategy:** 

Databases searched and dates covered:	CAB Abstracts on OVID platform 1910–2021 PubMed accessed on NCBI platform 1910–2021
Search strategy:	CAB Abstracts: (horse* or equi* or equus or pon*).mp. or exp horses/ or exp equus/ [mp=abstract, title, original title, broad terms, heading words, identifiers, cabicodes] (sinus* or paranasal or para-nasal or maxillary).mp. or exp sinuses/ or exp sinusitis/ [mp=abstract, title, original title, broad terms, heading words, identifiers, cabicodes] (CT or Computed tomography).mp. [mp=abstract, title, original title, broad terms, heading words, identifiers, cabicodes] (Radiograph* or xray* or x-ray* or radiol*).mp. [mp=abstract, title, original title, broad terms, heading words, identifiers, cabicodes] 1 and 2 and (3 or 4) PubMed: ** **Horse or horses or equine or equines or equus Sinus or sinusitis or paranasal or para-nasal or maxillary or teeth CT or computed tomography Radiography or xray or x-ray or radiology #1 and #2 and (#3 or #4)
Dates searches performed:	13 Nov 2021

**Exclusion / Inclusion Criteria:** 

Exclusion:	Non-relevant to the PICO. Non-English language. Study design was single case report. Non-empirical research. Duplicates.
Inclusion:	Relevant to PICO. Observational studies. Articles published in the English language. Retrospective studies.

**Search Outcome:** 

Database	Number of results	Excluded – Non-English Language	Excluded – Not relevant to PICO	Excluded – Single case report	Total relevant papers
CAB Abstracts	389	76	308	1	4
PubMed	369	15	350	0	4
Total relevant papers when duplicates removed					4

## Conflict of Interest

The author declares no conflicts of interest.

The authors acknowledge the contribution of Kirstie Pickles and Emma Shipman in the preparation of this manuscript.

## Disclaimer

Knowledge Summaries are a peer-reviewed article type which aims to answer a clinical question based on the best available current evidence. It does not override the responsibility of the practitioner. Informed decisions should be made by considering such factors as individual clinical expertise and judgement along with patient’s circumstances and owners’ values. Knowledge Summaries are a resource to help inform and any opinions expressed within the Knowledge Summaries are the author’s own and do not necessarily reflect the view of the RCVS Knowledge. Authors are responsible for the accuracy of the content. While the Editor and Publisher believe that all content herein are in accord with current recommendations and practice at the time of publication, they accept no legal responsibility for any errors or omissions, and make no warranty, express or implied, with respect to material contained within. For further information please refer to our Terms of Use.
